# Improved survival with enasidenib versus standard of care in relapsed/refractory acute myeloid leukemia associated with *IDH2* mutations using historical data and propensity score matching analysis

**DOI:** 10.1002/cam4.4182

**Published:** 2021-08-24

**Authors:** Stéphane de Botton, Joseph M. Brandwein, Andrew H. Wei, Arnaud Pigneux, Bruno Quesnel, Xavier Thomas, Ollivier Legrand, Christian Recher, Sylvain Chantepie, Mathilde Hunault‐Berger, Nicolas Boissel, Salem A. Nehme, Mark G. Frattini, Alessandra Tosolini, Roland Marion‐Gallois, Jixian J. Wang, Chris Cameron, Muhaimen Siddiqui, Brian Hutton, Gary Milkovich, Eytan M. Stein

**Affiliations:** ^1^ Departement d'hematologie Institut Gustave Roussy Villejuif France; ^2^ University of Alberta Edmonton Alberta Canada; ^3^ Alfred Hospital and Monash University Melbourne Australia; ^4^ CHU Bordeaux Hôpital du Haut Lévêque Bordeaux France; ^5^ CHU Lille Lille France; ^6^ Centre Hospitalier Lyon Sud Pierre Bénite France; ^7^ APHP Hôpital Saint‐Antoine Paris France; ^8^ CHU de Toulouse Université de Toulouse III Paul Sabatier Toulouse France; ^9^ CHU de Caen Caen France; ^10^ CHU Angers Angers France; ^11^ Hôpital Saint‐Louis Paris France; ^12^ Celgene International, a Bristol Myers Squibb Company Boudry Switzerland; ^13^ Bristol Myers Squibb Summit New Jersey USA; ^14^ Cornerstone Research Group Burlington Ontario Canada; ^15^ Ottawa Hospital Research Institute Ottawa Ontario Canada; ^16^ RJM Group LLC Crown Point Indiana USA; ^17^ Memorial Sloan Kettering Cancer Center New York New York USA

**Keywords:** acute myeloid leukemia, enasidenib, *IDH2* mutations, overall survival, standard of care

## Abstract

**Background:**

The present study evaluated the relative survival benefits associated with enasidenib and current standard of care (SoC) therapies for patients with relapsed/refractory (R/R) acute myeloid leukemia (AML) and an isocitrate dehydrogenase 2 (*IDH2)* mutation who are ineligible for hematopoietic stem cell transplantation (HSCT).

**Methods:**

Propensity score matching (PSM) analysis compared survival outcomes observed with enasidenib 100 mg daily in the phase I/II AG221‐C‐001 trial and SoC outcomes obtained from a real‐world chart review of patients in France.

**Results:**

Before matching, enasidenib (*n* = 195) was associated with numerically improved overall survival (OS) relative to SoC (*n* = 80; hazard ratio [HR], 0.82; 95% confidence interval [CI], 0.61–1.11). After matching and adjusting for covariates (*n* = 78 per group), mortality risk was significantly lower with enasidenib than with SoC (HR, 0.67; 95% CI, 0.47–0.97). The median OS was 9.26 months for enasidenib (95% CI, 7.72–13.24) and 4.76 months for SoC (95% CI, 3.81–8.21). Results remained robust across all sensitivity analyses conducted.

**Conclusions:**

PSM analyses indicate that enasidenib significantly prolongs survival relative to SoC among patients with R/R AML and an *IDH2* mutation who are ineligible for HSCT. Future prospective studies are needed to validate these findings using other data sources and to assess the comparative efficacy of enasidenib for other treatment outcomes.

## Graphical abstract

Overall survival was improved with enasidenib compared with SoC. Enasidenib may be an important advance in treatment for patients with R/R acute myeloid leukemia associated with IDH2 mutations who are ineligible for hematopoietic stem cell transplantation.

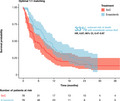

## 1 INTRODUCTION

Acute myeloid leukemia (AML) is a rare hematological malignancy that originates in the myeloid line of hematopoietic precursor cells.^1^, ^2^ Current global prevalence estimates range from approximately 0.6 to 11.0 per 100,000 persons, with most cases occurring in older individuals (median age at diagnosis: 67 years).^3^, ^4^ AML typically progresses rapidly, with an estimated 5‐year overall survival (OS) of 20%–27%.^5^, ^6^ The prognosis is particularly poor among patients with relapsed or refractory (R/R) disease, with a median OS of 3–6 months and an estimated 5‐year survival of 5%–10%.^7^, ^8^

Enasidenib is a first‐in‐class, selective inhibitor of mutant isocitrate dehydrogenase 2 (IDH2) proteins that is indicated for the treatment of adult patients with R/R AML with an *IDH2* mutation (m*IDH2*+) in the United States.^9^, ^10^, ^11^, ^12^, ^13^ In the phase I/II AG221‐C‐001 trial in patients with m*IDH2*+ R/R AML, enasidenib was associated with prolonged survival among responding patients.^14^, ^15^ To date, no published studies have directly compared treatment outcomes for enasidenib and current standard of care (SoC) therapies in patients with m*IDH2*+ R/R AML. The present study sought to indirectly compare the relative survival benefits associated with enasidenib and current SoC therapies in these patients using propensity score matching (PSM) analyses.

## 2 METHODS AND PATIENTS

### 2.1 Data sources and patient population

#### 2.1.1 Data sources

Data were obtained from the AG221‐C‐001 trial of enasidenib monotherapy and the French chart review (FCR) study of patients receiving SoC therapies.

##### AG221‐C‐001 trial

The AG221‐C‐001 trial (NCT01915498) was a phase I/II, multicenter, open‐label, dose escalation, and expansion trial that investigated the efficacy and safety of enasidenib in patients with advanced m*IDH2*+ hematological malignancies. The trial enrolled 345 patients (280 with R/R AML) aged ≥18 years who were ineligible for hematopoietic stem cell transplantation (HSCT), had a baseline Eastern Cooperative Oncology Group (ECOG) performance status score of 0–2, adequate renal and hepatic function, and a platelet count of ≥20,000/µl.

The AG221‐C‐001 trial included three phases: phase I dose escalation (*n* = 113), phase I expansion (*n* = 126), and phase II expansion (*n* = 106). Combining the phase I and phase II data sets produced a total of 214 patients with m*IDH2*+ R/R AML who received enasidenib 100 mg/day (i.e., the current indicated dose in the United States; phase I: *n* = 109; phase II: *n* = 105; data cutoff: 1 September 2017).

##### FCR study

The FCR study was a retrospective, observational, multicenter, chart‐review study of patients treated with SoC therapies (*n* = 103). The inclusion criteria used in the FCR study aligned with those used in the AG221‐C‐001 trial (i.e., patients aged ≥18 years with m*IDH2*+ R/R AML), as determined from health records or medical charts. The chart review was carried out at nine centers in France that had inpatient diagnostic and treatment facilities for patients diagnosed with AML between 1 September 2011 and 30 September 2016, belonged to a network of oncologists or hematologists treating patients with R/R AML, had been operational and treating patients with AML for ≥24 months, and had clinical records available for review. All patients identified with R/R AML and a m*IDH2*+ were screened for inclusion until >100 eligible patients were enrolled.

The SoC therapies administered included: 5‐azacitidine (37%), cytarabine‐containing regimens (22%), ′7 + 3′ chemotherapy (16%), methotrexate and mercaptopurine (1%), mercaptopurine (1%), decitabine (1%), clofarabine (1%), best supportive care (BSC; 11%), and other therapies (11%). Data were collected by a trained data reviewer to ensure accuracy, consistency, appropriateness, and completeness. Relevant data were also reviewed by the sponsor’s clinical research scientist and clinical research physician to ensure accurate chronology of patient data, and to confirm that definitions of therapy lines and disease progression aligned with those used in the AG221‐C‐001 trial.

Data in the FCR study were collected from the time of initial AML diagnosis. Conversely, patients in the AG221‐C‐001 trial were enrolled at various times after their first diagnosis of R/R AML and data were only collected after enrollment; the date of enasidenib treatment initiation was defined as the baseline date. Since the FCR study was a real‐world evidence study and lacked a clear date of treatment initiation, baseline (or the time origin [T0]; i.e., the time at which a patient entered the study) was defined as the initiation date of the last treatment line after the initial R/R disease diagnosis (Figure S1). This allowed comparability between the two study populations in terms of the number of treatment lines. For patients who did not receive any treatment after the first diagnosis of R/R AML, or who had missing information (e.g., no initiation date of the last treatment line after the last diagnosis of R/R AML), T0 was defined as the date of R/R diagnosis. Baseline characteristics of patients in the FCR study were assessed at T0. For example, the patient age collected in the FCR study was the age at initial AML diagnosis, but was adjusted to reflect the age at T0 for the present analyses.

### 2.2 Statistical methods

#### 2.2.1 Propensity score matching

After consultation with clinical experts, it was determined that the propensity score (PS) covariates with the greatest impact on treatment response in patients with m*IDH2*+ R/R AML were: prior history of HSCT (yes or no), age at baseline (<65 years vs. ≥65 years), number of prior lines of AML therapy (<2 vs. ≥2), cytogenetic risk profile (i.e., intermediate, poor, or failure/unevaluable; as per International Working Group cytogenetic risk criteria^14^), and history of myelodysplastic syndromes (MDS; yes vs. no) (Tables S1 and S2). Details regarding the calculation of the PS are provided in the supplementary information. For the primary analysis, patients from the enasidenib group were matched with patients from the SoC group using an optimal 1:1 matching algorithm based on the logit transform of the PS (LTPS) (Figure S2).^16^ As the number of patients in the SoC group (*n* = 78) was lower than the number of patients in the enasidenib group (*n* = 195), 1:1 optimal matching was considered more appropriate than other 1:1 matching algorithms.^17^ Other potential matching algorithms were evaluated in the sensitivity analyses as described below. In addition, the means, standard deviation (SD), and standardized mean difference (SMD) of each covariate were compared between treatment groups before and after matching.^18^, ^19^, ^20^

#### 2.2.2 Primary analysis

The main outcome of interest was OS, defined as the time from baseline (T0) to death from any cause. Kaplan–Meier plots were generated to summarize OS in the two treatment groups. A robust variance structure was incorporated in subsequent analyses to account for the use of PSM methods.^19^, ^21^, ^22^ The estimated hazard ratio (HR; and the corresponding confidence interval [CI]) for OS is the primary endpoint of this analysis. They were derived from Cox proportional hazards models that accounted for matching using robust variance estimators, where the time to death was regressed based on the treatment received. The appropriateness of the proportional hazards assumption necessary for Cox proportional hazards models were assessed based on visual inspection of the Kaplan–Meier plots, visual inspection of the Schoenfeld residuals plot, and a global test for non‐proportional hazards of any covariate (i.e., a two‐tailed *p* value of <0.05 indicated a violation of the assumption).^23^ A Cox model with multivariable regression adjustments using covariates identified by clinical experts (as mentioned above) was applied.^24^ In addition to the HR, the median survival time and survival at 3 and 12 months were reported to compare OS in the two treatment groups.

#### 2.2.3 Sensitivity analyses

Sensitivity analyses were conducted to assess the robustness of the primary analysis against the choice of matching algorithm. These included: using alternative applications of PS (i.e., full matching with caliper of width equal to 0.2 of the SD of the LTPS, inverse probability of treatment weighting [IPTW], and nearest neighbor matching), alternate weighting approaches (i.e., average treatment effect in the untreated, average treatment effect in the treated, and average treatment effect in the entire sample), as well as analyses stratified by matched pairs.^25^ In sensitivity analyses using PS weighting methods instead of matching, weighted Cox models were used. The following sensitivity analyses were also conducted: (1) adding ECOG as a sixth covariate to the primary analysis and (2) using optimal 1:1 matching based upon LTPS by treating age as a continuous variable for both PS estimation and multivariable modeling in the Cox proportional hazards analysis. Additional variables were not considered for sensitivity analyses because of the missingness of data.

All statistical analyses were performed using R software (version 3.4.2; R Core Team), through the application of the ‘MatchIt’^26^ (version 3.0.2) and ‘optmatch’^27^ (version 0.9‐11) packages. Statistical significance was defined using a two‐tailed *p* value of <0.05, and all comparisons between groups were reported with the respective HR and associated 95% CI, which incorporated robust variance estimators.

## 3 RESULTS

### 3.1 Primary analysis population

The primary analysis population included patients with m*IDH2*+ R/R AML. Notably, the AG221‐C‐001 trial excluded patients for whom potentially curative anticancer therapy (i.e., HSCT) was available at the time of enrollment. However, patients could become eligible to undergo HSCT during the course of the trial. In contrast, the FCR study did not have such an exclusion criterion, given its retrospective and observational nature. To ensure a valid estimate of comparative efficacy and alignment between the patient populations, the PSM analysis population excluded patients who underwent HSCT after baseline in both data sources (*n* = 19 for the AG221 C‐001 trial and *n* = 23 for the FCR study). As the estimation of PS requires complete information for all covariates, the primary analysis population excluded patients with missing data for any of the covariates assessed. After the exclusion of patients with subsequent HSCT or missing data, the primary analysis population consisted of 195 patients treated with enasidenib in the AG221 C‐001 trial and 78 patients treated with SoC therapies in the FCR study.

### 3.2 Pre‐match and post‐match balance between treatment groups

After exclusion of patients who underwent HSCT after baseline, the pre‐match population included 195 patients treated with enasidenib in the AG221‐C‐001 trial and 80 patients treated with SoC therapies in the FCR study (Table S3). After matching for all possible covariates and excluding patients with missing data, a total of 156 patients were included in the primary analysis (*n* = 78 for each treatment group). For each covariate, the SMD was compared before and after matching to determine if the PSM methods resulted in sufficient balance between groups (Table S3). A decrease in the SMD indicated an improved balance between the two groups, whereas a threshold of ≥0.10 indicated a potentially relevant remaining imbalance between the groups.^28^

### 3.3 Pre‐matching comparison

Before matching, the treatment groups had a similar proportion of patients with prior MDS (enasidenib: 22%; SoC: 20%) and age ≥65 years (enasidenib: 64%; SoC: 62%). However, considerable differences were observed for the number of prior treatment lines, prior HSCT, and cytogenetic risk profile, as indicated by an SMD of ≥0.10 for each covariate. Fewer patients in the enasidenib group had prior HSCT (enasidenib: 14%; SoC: 24%), and more patients had previously received ≥2 prior lines of therapy (enasidenib: 53%; SoC: 33%). Cytogenetic risk was also more unfavorable in the enasidenib group, with fewer intermediate‐risk patients (enasidenib: 49% vs. SoC: 84%), and more poor‐risk patients (enasidenib: 27% vs. SoC: 6%) or failure/unevaluable patients (enasidenib: 24% vs. SoC: 10%) (Table S3).

### 3.4 Post‐matching comparison

The enasidenib and SoC groups were well balanced after 1:1 optimal matching, with most SMDs improving after matching (Table S3). Density plots of the PS estimates pre‐ and post‐matching are depicted in Figure S3. The post‐matching PS distribution was nearly identical between treatment groups.

### 3.5 Primary analysis

**FIGURE 1:**
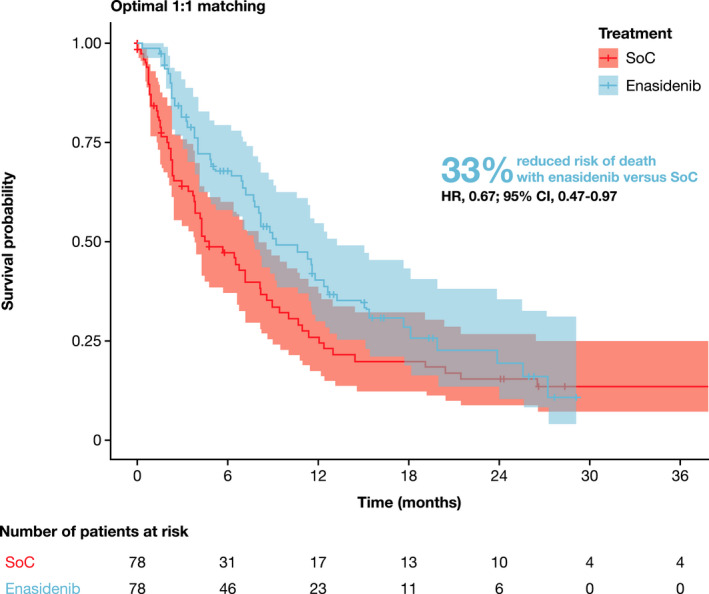
Kaplan–Meier estimate of overall survival (OS) for optimal 1:1 PS‐matched sample of patients receiving enasidenib (AG221‐C‐001 trial) and standard of care (SoC) (French chart review [FCR] study)

**FIGURE 2:**
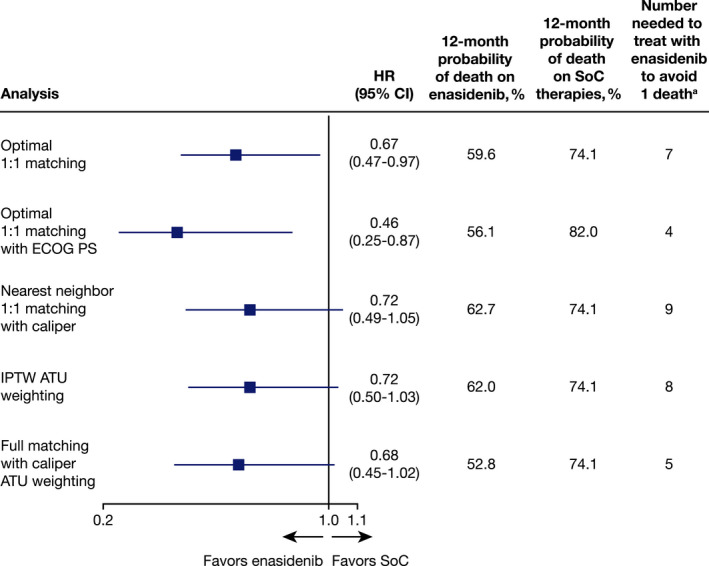
Comparison of overall survival (OS) as determined through various PSM algorithms

Before matching, enasidenib was associated with numerically prolonged OS relative to SoC (HR, 0.82; 95% CI, 0.61–1.11; Table S4). After matching, enasidenib was associated with significantly prolonged OS relative to SoC (HR, 0.67; 95% CI, 0.47–0.97; Figure 1). The median OS was 9.26 months for enasidenib (95% CI, 7.72–13.24) and 4.76 months for SoC (95% CI, 3.81–8.21). The estimated 3‐month survival rates for enasidenib and SoC were 82% and 64%, respectively, with estimated 12‐month survival rates of 40% and 26%, respectively (Figure 2).

### 3.6 Sensitivity analyses

Multiple sensitivity analyses were conducted to assess the robustness of the primary OS analysis, including alternative applications of PS and weighting approaches, as well as strata‐adjusted analyses. Across all analyses, effect estimates remained either numerically or statistically significantly in favor of enasidenib (Figure 2, Table S4).

## 4 DISCUSSION

In the absence of head‐to‐head randomized clinical trial data, PSM methods^17^ can be used to leverage individual patient data to estimate the treatment effect between two interventions from separate studies, as shown in an analysis by Takahashi and colleagues.^29^ In the present study, a PSM analysis was conducted to compare survival outcomes for patients with m*IDH2*+ R/R AML treated with enasidenib from the AG221‐C‐001 trial and SoC therapies from the FCR study. After matching, treatment with enasidenib was associated with a statistically significant reduction in mortality risk compared with SoC (HR, 0.67; 95% CI, 0.47–0.97). The results remained robust across a range of sensitivity analyses (i.e., with HR values ranging from 0.46 to 0.72), suggesting consistent survival benefits for enasidenib beyond those provided by current SoC therapies. The estimated 12‐month probability of survival remained significantly higher in patients receiving enasidenib (37.3%–47.2%) compared with SoC therapies (18%–25.9%) through sensitivity analyses for matching, IPTW, nearest neighbor matching, and ECOG performance status score.

Current treatment options for patients with R/R AML are limited, and there is a substantial unmet need for effective, well‐tolerated therapies.^2^, ^30^, ^31^, ^32^, ^33^ This is the first study conducted to compare enasidenib and SoC therapies for the treatment of patients with m*IDH2*+ R/R AML, and results suggest that enasidenib significantly prolongs survival relative to SoC therapies. These findings provide evidence in support of enasidenib as an important treatment option for patients with m*IDH2*+ R/R AML.

A recent study by Largeaud and colleagues evaluated real‐world treatment outcomes among patients with m*IDH2*+ R/R AML who received treatment before the introduction of IDH2 inhibitors.^34^ Available treatment options were associated with similar survival rates to those observed in patients treated with enasidenib in the AG221‐C‐001 trial.^14^, ^15^ However, it is important to note that the baseline definition used in the study by Largeaud and colleagues^34^ differed from the present analyses of the AG221‐C‐001 trial and the FCR study, meaning that survival was not calculated from the same line of therapy. The use of salvage treatment (including intensive treatment) was excluded from the present analyses but was allowed in the Largeaud study,^34^ which may also have led to the improved survival rates.

An estimated 12% of enasidenib‐treated patients with m*IDH2*+ AML eventually develop isocitrate dehydrogenase differentiation syndrome (IDH‐DS).^34^ The syndrome is difficult to diagnose, as it is not characterized by a single symptom but rather a collection of symptoms that can mimic those of leukemic progression or other comorbidities. IDH‐DS has the potential to be life‐threatening, but can be managed with appropriate care.^35^ Patients treated with enasidenib should be monitored for IDH‐DS, to ensure prompt diagnosis and subsequent treatment.

There are several potential limitations to consider when interpreting the findings of this study. First, certain factors could not be included for PS estimation or subsequent statistical adjustments because of limited available data, such as the duration of first response. However, the most comprehensive set of available covariates was included, considering the prognostic significance rankings of the four clinical experts and the availability of patient information from the study data sources. This resulted in the majority of the most highly ranked prognostic factors being included in the analyses. Secondly, criticisms of analyses based on PSM techniques, such as incomplete sampling from the treatment and control groups, have previously been reported.^17^ To address this limitation, this study included a comprehensive set of sensitivity analyses using well‐established PSM methods. In addition to the primary analysis, full matching and IPTW methods were conducted so that all patients in the pre‐match population could be retained for analysis. This was important, as it is rare for the control group to include fewer patients than the intervention group, as was the case in this study. Thirdly, patients with missing data were excluded from the analyses (i.e., imputations for missing data were not performed). However, none of the patients in the AG221‐C‐001 trial and only two patients in the FCR study were excluded because of missing data; therefore, the impact on the overall findings was likely minimal. Lastly, as is the case with any non‐randomized study, analyses employing multivariable modeling or matching techniques cannot adjust for unknown confounding variables that may influence patient outcomes. Of note, arbitrarily defining T0 as the start of the last treatment in the FCR study may have influenced OS in the SoC group. Future research adjusting for additional factors not captured in this study should be completed to further understand how these variables might impact clinical outcomes among patients with m*IDH2*+ R/R AML.

In conclusion, the present findings suggest that enasidenib offers greater survival benefit than SoC therapies among patients with m*IDH2*+ R/R AML who are ineligible for HSCT. Future studies are needed to validate these findings using other data sources, and to assess the comparative efficacy of enasidenib for other treatment outcomes.

## CONFLICT OF INTERESTS

SdB has received research funding and served on an advisory board for Agios; participated in speakers’ bureaus and served on advisory boards for AbbVie, Bristol Myers Squibb, and Janssen; served on an advisory board and provided consultancy to Pierre Fabre; and served on advisory boards for Astellas, Bayer, Daiichi‐Sankyo, Forma, Novartis, Pfizer, Servier, and Syros. JMB has served on advisory boards and received honoraria from Bristol Myers Squibb, Jazz Pharmaceuticals, Novartis, Otsuka, Pfizer, and Teva; and received research funding from Bristol Myers Squibb. AHW has received research funding and honoraria and served on advisory boards for AbbVie, Bristol Myers Squibb, Novartis, and Servier. AP has received honoraria from AbbVie, Amgen, Astellas, Bristol Myers Squibb, Daiichi‐Sankyo, Jazz Pharmaceuticals, Novartis, Pfizer, and Roche. BQ has received honoraria from Astellas, Celyad, Novartis, and Sunesis. CR has served on advisory boards for AbbVie, Janssen, MacroGenics, and Pfizer; served on advisory boards for and received research funding from Astellas, Bristol Myers Squibb, Daiichi‐Sankyo, Jazz Pharmaceuticals, Novartis, and Sunesis; and received research funding from Agios, Amgen, Chugai, and MaaT Pharma. MH‐B has interests in AbbVie, Astellas, Daiichi‐Sankyo, and Sunesis. NB has received honoraria from Amgen, Ariad, Jazz Pharmaceuticals, Pfizer, Shire, and Servier; and served on advisory boards for Bristol Myers Squibb. SAN, MGF, AT, RM‐G, and J(J)W are employees of Bristol Myers Squibb. CC is an employee and shareholder of EVERSANA Life Sciences Services Inc. MS is an employee of EVERSANA Life Sciences Services Inc. BH is a methodological advisor for EVERSANA Life Sciences Services Inc. GM has provided consultancy services to Bristol Myers Squibb. EMS has received consulting fees from Agios, Amgen, Astellas, Bristol Myers Squibb, Daiichi‐Sankyo, Genentech, Novartis, PTC Therapeutics, Seattle Genetics, and Syros. XT, OL, and SC have no conflicts of interest to disclose.

## ETHICAL STATEMENT

BMS is the manufacturer of enasidenib and sponsored the trial and chart review that were used in this study. The concept and design for PSM analyses underwent internal BMS approval and EVERSANA was provided access to anonymized data from the AG221‐C‐001 trial and France Chart Review to run the analyses after approval. AG221‐C‐001 was registered with Clinicaltrials.gov (NCT01915498). The AG221‐C‐001 protocol received approval in 24 study locations from their local institutional review board or independent ethics committees. The France Chart Review study protocol did not require any submission or approval since Aixial has a global commitment to follow processes required by the Commission Nationale de l’Informatique et des Libertes (CNIL) called MR003. All patients in the AG221‐C‐001 trial were required to provide written informed consent before any study‐related procedures were performed.

## PRECIS

Results from this PSM analysis indicate that enasidenib significantly prolongs OS relative to the current SoC for patients with relapsed/refractory AML and *IDH2* mutations who are ineligible for HSCT.

## Supporting information

Supplementary Material

## Data Availability

The data that support the findings of this study are available upon request from the corresponding author. The data are not publicly available due to privacy or ethical restrictions.

## References

[1] Döhner H, Weisdorf DJ, Bloomfield CD. Acute myeloid leukemia. N Engl J Med. 2015;373(12):1136‐1152. 26376137 10.1056/NEJMra1406184

[2] De Kouchkovsky I, Abdul‐Hay M. Acute myeloid leukemia: a comprehensive review and 2016 update. Blood Cancer J. 2016;6(7):e441. 27367478 10.1038/bcj.2016.50 PMC5030376

[3] Lubeck DP, Danese M, Jennifer D, Miller K, Richhariya A, Garfin PM. Systematic literature review of the global incidence and prevalence of myelodysplastic syndrome and acute myeloid leukemia. Blood. 2016;128(22):5930.

[4] Kell J. Considerations and challenges for patients with refractory and relapsed acute myeloid leukaemia. Leuk Res. 2016;47:149‐160. 27371910 10.1016/j.leukres.2016.05.025

[5] Cancer Research UK . Acute myeloid leukaemia (AML) incidence statistics. 2015. Accessed May 2018. http://www.cancerresearchuk.org/health‐professional/cancer‐statistics/statistics‐by‐cancer‐type/leukaemia‐aml/incidence

[6] National Cancer Institute . Cancer stat facts: leukemia—acute myeloid leukemia (AML). Accessed May 2018. https://seer.cancer.gov/statfacts/html/amyl.html

[7] Oran B, Weisdorf DG. Survival for older patients with acute myeloid leukemia: a population‐based study. Haematologica. 2012;97(12):1916‐1924. 22773600 10.3324/haematol.2012.066100 PMC3590098

[8] Walter RB, Estey EH. Management of older or unfit patients with acute myeloid leukemia. Leukemia. 2015;29(4):770‐775. 25005246 10.1038/leu.2014.216 PMC4289127

[9] Gross S, Cairns RA, Minden MD, et al. Cancer‐associated metabolite 2‐hydroxyglutarate accumulates in acute myelogenous leukemia with isocitrate dehydrogenase 1 and 2 mutations. J Exp Med. 2010;207:339‐344. 20142433 10.1084/jem.20092506 PMC2822606

[10] Losman JA, Looper R, Koivunen P, et al. (R)‐2‐hydroxyglutarate is sufficient to promote leukemogenesis and its effects are reversible. Science. 2013;339(6127):1621‐1625. 23393090 10.1126/science.1231677 PMC3836459

[11] Figueroa ME, Abdel‐Wahab O, Lu C, et al. Leukemic IDH1 and IDH2 mutations result in a hypermethylation phenotype, disrupt TET2 function, and impair hematopoietic differentiation. Cancer Cell. 2010;18(6):553‐567. 21130701 10.1016/j.ccr.2010.11.015 PMC4105845

[12] Stein EM. IDH2 inhibition in AML: finally progress? Best Pract Res Clin Haematol. 2015;28(2–3):112‐115. 26590767 10.1016/j.beha.2015.10.016

[13] United States Food and Drug Administration , Updated 2017. Accessed January 2019. https://www.fda.gov/drugs/informationondrugs/approveddrugs/ucm569482.htm

[14] Stein EM, DiNardo CD, Pollyea DA, et al. Enasidenib in mutant IDH2 relapsed or refractory acute myeloid leukemia. Blood. 2017;130(6):722‐731. 28588020 10.1182/blood-2017-04-779405 PMC5572791

[15] Stein EM, DiNardo CD, Fathi AT, et al. Molecular remission and response patterns in patients with mutant‐IDH2 acute myeloid leukemia treated with enasidenib. Blood. 2019;133(7):676‐687. 30510081 10.1182/blood-2018-08-869008 PMC6384189

[16] Stuart EA. Matching methods for causal inference: a review and a look forward. Stat Sci. 2010;25(1):1‐21. 20871802 10.1214/09-STS313 PMC2943670

[17] Austin PC. An introduction to propensity score methods for reducing the effects of confounding in observational studies. Multivariate Behav Res. 2011;46(3):399‐424. 21818162 10.1080/00273171.2011.568786 PMC3144483

[18] Austin PC, Stuart EA. Moving towards best practice when using inverse probability of treatment weighting (IPTW) using the propensity score to estimate causal treatment effects in observational studies. Stat Med. 2015;34(28):3661‐3679. 26238958 10.1002/sim.6607 PMC4626409

[19] Austin PC, Schuster T. The performance of different propensity score methods for estimating absolute effects of treatments on survival outcomes: a simulation study. Stat Methods Med Res. 2016;25(5):2214‐2237. 24463885 10.1177/0962280213519716 PMC5051602

[20] Faria R, Alava MH, Manca A, Wailoo AJ. NICE DSU Technical Support Document 17: the use of observational data to inform estimates of treatment effectiveness in technology appraisal: methods for comparative individual patient data. 2015. Accessed July 2019. http://nicedsu.org.uk/wp‐content/uploads/2016/03/TSD17‐DSU‐Observational‐data‐FINAL.pdf

[21] Gayat E, Resche‐Rigon M, Mary JY, Porcher R. Propensity score applied to survival data analysis through proportional hazards models: a Monte Carlo study. Pharm Stat. 2012;11(3):222‐229. 22411785 10.1002/pst.537

[22] Austin PC. The use of propensity score methods with survival or time‐to‐event outcomes: reporting measures of effect similar to those used in randomized experiments. Stat Med. 2014;33(30):1242‐1258. 24122911 10.1002/sim.5984 PMC4285179

[23] Dunkler D, Ploner M, Schemper M, Heinze G. Weighted Cox regression using the R package coxphw. J Stat Softw. 2018;84(2):1‐26.30450020

[24] Funk MJ, Westreich D, Wiesen C, Stürmer T, Brookhart MA, Davidian M. Doubly robust estimation of causal effects. Am J Epidemiol. 2011;173(7):761‐767. 21385832 10.1093/aje/kwq439 PMC3070495

[25] Austin PC, Stuart EA. The performance of inverse probability of treatment weighting and full matching on the propensity score in the presence of model misspecification when estimating the effect of treatment on survival outcomes. Stat Methods Med Res. 2017;26(4):1654‐1670. 25934643 10.1177/0962280215584401 PMC5564952

[26] Ho DE, Imai K, King G, Stuart EA. MatchIt: nonparametric preprocessing for parametric causal inference. J Stat Soft. 2011;42(8):1‐28. Accessed October 2019. https://imai.fas.harvard.edu/research/files/matchit.pdf

[27] Optmatch . Package ‘optmatch’. Version 0.9‐12. Accessed October 2019. https://cran.r‐project.org/web/packages/optmatch/optmatch.pdf

[28] Austin PC. Some methods of propensity‐score matching had superior performance to others: results of an empirical investigation and Monte Carlo simulations. Biom J. 2009;51(1):171‐184. 19197955 10.1002/bimj.200810488

[29] Takahashi K, Kantarjian H, Yang Y, et al. A propensity score matching analysis of dasatinib and nilotinib as a frontline therapy for patients with chronic myeloid leukemia in chronic phase. Cancer. 2016;122(21):3336‐3343. 27509035 10.1002/cncr.30197 PMC5073019

[30] Wiese M, Daver N. Unmet clinical needs and economic burden of disease in the treatment landscape of acute myeloid leukemia. Am J Manag Care. 2018;24(16 Suppl):S347‐S355. 30132678

[31] Lyle L, Daver N. Current and emerging therapies for patients with acute myeloid leukemia: a focus on MCL‐1 and the CDK9 pathway. Am J Manag Care. 2018;24:S356‐S365. 30132679

[32] Pemmaraju N, Kantarjian H, Garcia‐Manero G, et al. Improving outcomes for patients with acute myeloid leukemia in first relapse: a single center experience. Am J Hematol. 2015;90(1):27‐30. 25251041 10.1002/ajh.23858 PMC4276516

[33] DiNardo CD, Stein EM. SOHO state of the art update and next questions: IDH therapeutic targeting in AML. Clin Lymphoma Myeloma Leuk. 2018;18(12):769‐772. 30416011 10.1016/j.clml.2018.10.007

[34] Largeaud L, Bérard E, Bertoli S, et al. Outcome of AML patients with IDH2 mutations in real world before the era of IDH2 inhibitors. Leuk Res. 2019;81:82‐87. 31055247 10.1016/j.leukres.2019.04.010

[35] Fathi AT, DiNardo CD, Kline I, et al. Differentiation syndrome associated with enasidenib, a selective inhibitor of mutant isocitrate dehydrogenase 2: analysis of a phase 1/2 study. JAMA Oncol. 2018;4(8):1106‐1110. 29346478 10.1001/jamaoncol.2017.4695 PMC5885269

