# Supplementary material for: Improved survival with enasidenib versus standard of care in relapsed/refractory acute myeloid leukemia associated with IDH2 mutations using historical data and propensity score matching analysis
Source: Cancer Med. 2021 Aug 24;10(18):6336–43. doi: 10.1002/cam4.4182 (PMC8446562; doi:10.1002/cam4.4182)
Supplement: Supplementary file 1 — Supplementary Material [file CAM4-10-6336-s001.docx]

# Supplementary information

## Covariates selected for propensity score (PS)

Four clinical advisors from different geographical locations (the United States, Canada, Australia, and France) were consulted about which prognostic factors would have the largest impact on treatment response in patients with relapsed or refractory (R/R) acute myeloid leukemia (AML) with an IDH2 mutation (mIDH2+). Each clinician independently ranked the prognostic factors (Table S1) and aggregate rankings with mean rank orders were calculated. Aggregated rankings from clinicians and the availability of prognostic factors in the AG221 C-001 trial and French Chart Review (FCR) study were considered collectively (Table S2) to determine the final set of prognostic factors to be used for the analyses.

## Calculation of PS

For the primary analysis, PS were defined as the conditional probability of each patient being assigned to the standard of care (SoC) group (ie, group with the smaller sample size). PS were estimated by fitting a multivariable logistic regression that included the final set of prognostic factors as covariates (ie, predictor variables) and the treatment received (ie, enasidenib or SoC therapies) as the dependent variable. All covariates were evaluated based on the respective baseline dates for the AG221-C-001 trial and the FCR study. One sensitivity analysis included age as a continuous variable rather than a categorical variable. After fitting the logistic regression model, the logit transformed propensity score (LTPS) for all patients was stored for subsequent use in PS analyses.^21^ Following estimation of LTPS, the degree of overlap in patient PS was assessed through density plots for LTPS estimates in each treatment group.

SUPPLEMENTARY TABLE S1 Summary of clinical advisor rank ordering of prognostic factors for OS

| Prognostic factor rank | Clinician advisor #1 ordering | Clinician advisor #2 ordering | Clinician advisor #3 ordering | Clinician advisor #4 ordering |
| --- | --- | --- | --- | --- |
| 1 | NCCN/ELN risk classification | Age | Duration of first response | Age |
| 2 | First CR <12 months | History of stem cell transplant | Lines of therapy | Cytogenetic risk profile |
| 3 | History of stem cell transplant | Lines of AML therapy received | History of stem cell transplant | Lines of AML therapy |
| 4 | Prior MDS | Cytogenetic risk profile | Cytogenetic risk profile | Performance status (ECOG) |
| 5 | Performance status (ECOG) | Duration of first response/first CR <12 months | Age | History of stem cell transplant |
| 6 | Lines of AML therapy received | Performance status (ECOG) | WBC count | Duration of first response |
| 7 | Age | *FLT3* mutation status | *FLT3* mutation status | Prior MDS |
| 8 | WBC count | Prior MDS | Prior MDS | *FLT3*-ITD mutation status |
| 9 | Creatinine clearance | Platelet count | Creatinine clearance | Creatinine clearance |
| 10 | *IDH2* mutation location | Bone marrow blast | Bone marrow blast | WBC count |
| 11 |  | *IDH2* mutation location | Platelet count |  |
| 12 |  | WBC count |  |  |
| 13 |  | Hemoglobin count |  |  |
| 14 |  | WHO AML classification |  |  |
| 15 |  | Creatinine clearance |  |  |

Abbreviations: AML**,** acute myeloid leukemia; CR**,** complete response; ECOG**,** Eastern Cooperative Oncology Group; ELN**,** European Leukemia Network; *FLT3***,** FMS-related tyrosine kinase 3; *FLT3*-ITD**,** *FLT3* internal tandem duplication; *IDH2***,** isocitrate dehydrogenase 2; MDS**,** myelodysplastic syndromes; NCCN**,** National Comprehensive Cancer Network; OS, overall survival; WBC**,** white blood cell; WHO**,** World Health Organization.

SUPPLEMENTARY TABLE S2 Clinician rankings for OS and prognostic factor availability from data sources

| Mean rank ordering | Prognostic factors | Data available in the FCR study? | Data available in the AG221-C-001 trial? | Significant number of missing values in both or either dataset? |
| --- | --- | --- | --- | --- |
| 1 | History of stem cell transplant | Y | Y | N |
| 2 | Lines of AML therapy | Y | Y | N |
| 3 | Age | Y | Y | N |
| 4 | Duration of first response/  first CR <12 months | Y | Y | Y |
| 5 | Cytogenetic risk profile | Y | Y | N |
| 6 | Prior MDS | Y | Y | N |
| 7 | Performance status (ECOG) | Y | Y | Y |
| 8 | *FLT3* mutation status | Y | N | Y |
| 9 | WBC count at baseline | Y^a^ | Y | Y |
| 10 | Creatinine clearance at baseline | N | Y | Y |
| 11 | NCCN/ELN risk classification | N | N | Y |
| 12 | *IDH2* mutation location | N | Y | Y |
| 13 | Platelet count at baseline | Y^a^ | Y | Y |
| 14 | Bone marrow blast at baseline | Y^a^ | Y | Y |
| 15 | Hemoglobin count at baseline | Y^a^ | Y | Y |

Abbreviations: AML**,** acute myeloid leukemia; CR**,** complete response; ECOG**,** Eastern Cooperative Oncology Group; ELN**,**European LeukemiaNet; FCR, French Chart Review; *FLT3***,** FMS-related tyrosine kinase 3; *IDH2***,** isocitrate dehydrogenase 2; MDS**,**myelodysplastic syndromes; N**,**no; NCCN**,** National Comprehensive Cancer Network; OS**,** overall survival; WBC**,**white blood cell; Y**,** yes.

^a^ Although some laboratory data were available and collected, they were rarely obtained at a time corresponding to baseline, and were therefore considered missing at baseline.

SUPPLEMENTARY TABLE S3 Overview of group demographic balance before and after matching

| Covariates | Pre-matching characteristics | | | Post-matching characteristics^a^ (optimal 1:1 matching) | | |
| --- | --- | --- | --- | --- | --- | --- |
|  | SoC group | Enasidenib group | SMD^b^ | SoC group | Enasidenib group | SMD^b^ |
| N | 80 | 195 | NA | 37^c^ | 37 | NA |
| Prior HSCT, % | 24 | 14 | **0.26** | 22 | 22 | <0.001 |
| ≥2 prior lines of AML therapy, % | 33 | 53 | **0.42** | 30 | 22 | **0.18** |
| Age ≥65 years, % | 62 | 64 | 0.03 | 70 | 76 | **0.12** |
| Cytogenetic risk profile, % |  |  |  |  |  |  |
| Intermediate | 84 | 49 | **0.78** | 89 | 84 | **0.16** |
| Poor | 6 | 27 | **0.57** | 8 | 14 | **0.17** |
| Failure/ unevaluable | 10 | 24 | **0.38** | 3 | 3 | <0.001 |
| Prior MDS, % | 20 | 22 | 0.04 | 16 | 16 | <0.001 |
| ECOG status, % |  |  |  |  |  |  |
| 0 | 18 | 23 | **0.12** | 17 | 24 | **0.20** |
| 1 | 59 | 62 | 0.06 | 59 | 59 | <0.001 |
| 2 | 23 | 15 | **0.19** | 24 | 17 | **0.20** |
| Proportion with SMD ≥0.10, % | 70 | | | 60 | | |
| Mean SMD | 0.285 | | | 0.103 | | |

Abbreviations: AML, acute myeloid leukemia; ECOG, Eastern Cooperative Oncology Group; HSCT, hemopoietic stem cell transplant; MDS, myelodysplastic syndromes; NA, not available/applicable; PSM, propensity score matching; SMD, standardized mean difference; SoC, standard of care.

^a^ PSM algorithm was based on five covariates (history of HSCT at baseline, prior lines of therapy, age, cytogenetic risk profile, and history of MDS).
^b^ SMD greater than 0.10 are shown in bold, which express imbalances between the groups.
^c^ Due to missing ECOG status data, fewer patients were eligible for inclusion in the 1:1 matching. Two patients from the SoC group were removed due to missing covariate values.

SUPPLEMENTARY TABLE S4 Summary of comparisons of OS, enasidenib versus SoC

| **Analysis performed** | **Number of deaths** | **Number of patients** | **HR (95% CI), enasidenib vs SoC** |
| --- | --- | --- | --- |
| Primary analysis | | | |
| Adjusted HR, Optimal 1:1 matching | 115 | 156 | MVA: 0.67  (0.47-0.97)  **Strata: 0.42  (0.28-0.64)** |
|  |  |  |  |
| Pre-matched analyses: primary analysis population | | | |
| Unadjusted HR (prior to PSM, all patients) | 213 | 274 | 0.82 (0.61-1.11) |
|  |  |  |  |
| Unadjusted HR (prior to PSM, excluding those with missing covariates) | 212 | 273 | 0.84 (0.62-1.13) |
|  |  |  |  |
| Adjusted HR (prior to PSM, excluding those with missing covariates) | 212 | 273 | 0.75 (0.54-1.04) |
|  |  |  |  |
| Reference case: participants with HSCT after baseline not excluded | | | |
| Unadjusted HR (prior to PSM, all patients) | 231 | 317 | 1.05 (0.79-1.39) |
| Unadjusted HR (prior to PSM, excluding those with missing covariates) | 230 | 316 | 1.07 (0.80-1.42) |
| Adjusted HR (prior to PSM, excluding those with missing covariates) | 230 | 316 | 0.92 (0.68-1.25) |
| Sensitivity analyses: optimal matching, greedy matching, and IPTW | | | |
| Optimal 1:1 matching, model adjusted (age as a continuous variable) | 115 | 156 | 0.75 (0.52-1.08) |
| Optimal 1:1 matching, patients with HSCT after baseline included and censored at time of transplant | 125 | 204 | 0.79 (0.55-1.12) |
| Optimal 1:1 matching, model adjusted (landmark analysis – 11 days)^a^ | 110 | 148 | 0.72 (0.50-1.05) |
| Optimal 1:1 matching, model adjusted (landmark analysis – 1 month)^b^ | 92 | 128 | 0.76 (0.51-1.14) |
| Nearest neighbor 1:1 matching (caliper 0.2 SD LTPS) | 111 | 148 | 0.72 (0.49-1.05) |
|  |  |  |  |
| IPTW: ATU weighting | 212 | 273 | 0.72 (0.50-1.03) |
| IPTW: ATT weighting | 212 | 273 | 0.75 (0.48-1.19) |
| IPTW: ATE weighting | 212 | 273 | 0.74 (0.50-1.11) |
| Sensitivity analyses: full matching, caliper 0.2 SD LTPS | | | |
| ATU weighting | 182 | 243 | MVA: 0.68  (0.45-1.02)  **Strata: 0.45  (0.31-0.67)** |
| ATT weighting | 182 | 243 | MVA: 0.79  (0.47-1.32)  **Strata: 0.66  (0.42-1.01)** |
| ATE weighting | 182 | 243 | MVA: 0.78  (0.50-1.24)  **Strata: 0.59  (0.41-0.86)** |

Abbreviations: AML, acute myeloid leukemia; ATE, average treatment effect in the entire sample; ATT, average treatment effect in the treated; ATU, average treatment effect in the untreated; CI, confidence interval; HR, hazard ratio; HSCT, hematopoietic stem cell transplant; IPTW, inverse probability treatment weighting; MVA, multivariable adjusted; OS, overall survival; PSM, propensity score matching; LTPS, logit transformed propensity score; SD, standard deviation; SoC, standard of care.

HR <1 favor enasidenib 100 mg/day, and statistically significant differences are shown in bold font. Results denoted as “MVA” indicate HR derived from Cox proportional hazards analyses that included multivariable adjustments for age, prior HSCT, cytogenetic risk, history of MDS and number of prior lines of AML therapy. Results denoted as “Strata” indicate HR derived from Cox proportional hazards analyses that included an adjustment for propensity score-matched strata of treated and control patients.

^a^ Participants who died or were censored prior to 10 or less were removed from the analysis population

^b^ Participants who died or were censored prior to 1 month were removed from the analysis population

SUPPLEMENTARY FIGURE S1 Determination of baseline (or time origin [T0]) for the FCR study^a^


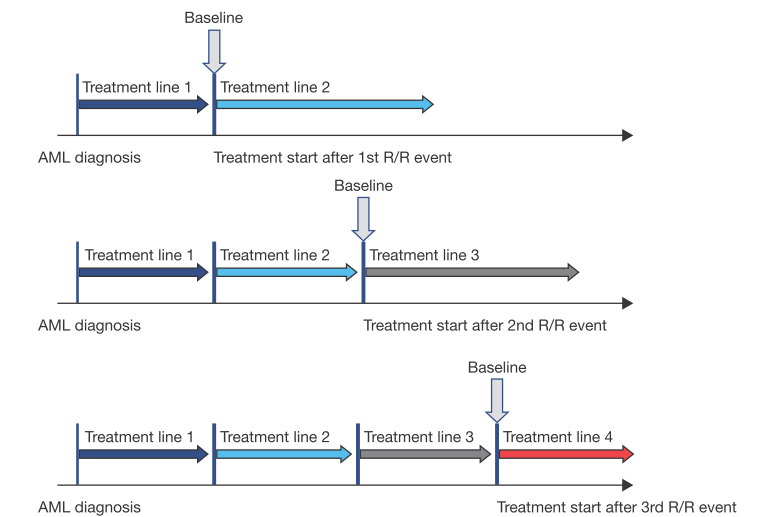


AML, acute myeloid leukemia; FCR, French Chart Review; R/R, relapsed or refractory

^a^ Baseline was set at the start of the most recent therapy line after AML was considered to be R/R, or at the time of R/R determination for patients who received no subsequent therapy line

SUPPLEMENTARY FIGURE S2 Overview of conventional PSM methods


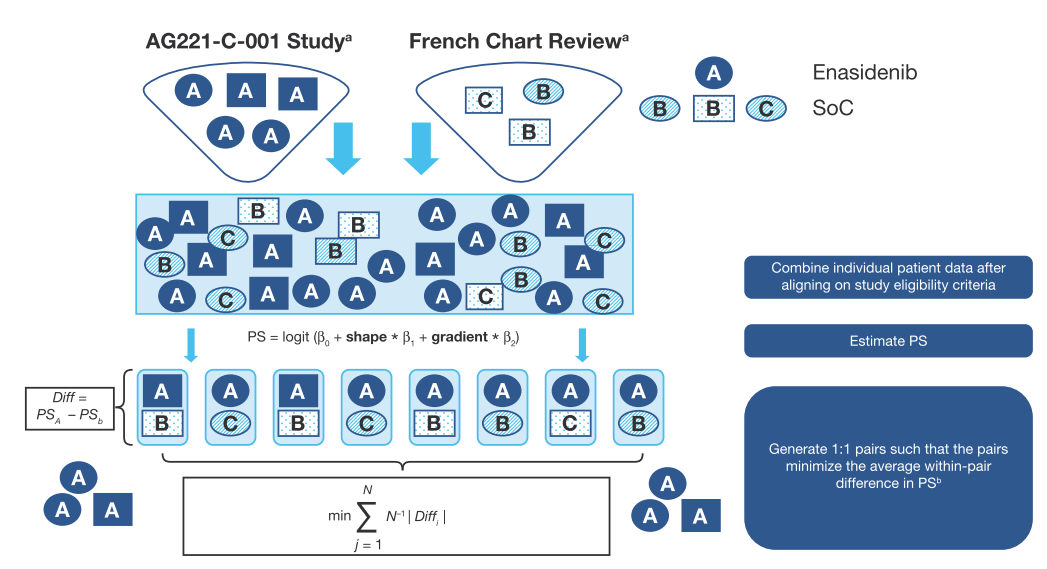
 Diff, difference; PS, propensity score; PSM, propensity score matching; SoC, standard of care

^a^ Various shapes, sizes, and gradients represent different patient characteristics
^b^ Optimal 1:1 matching was used to allow inclusion of all SoC patients

SUPPLEMENTARY FIGURE S3 Distribution of propensity scores by treatment group. A, Pre-matching and B, Post-matching


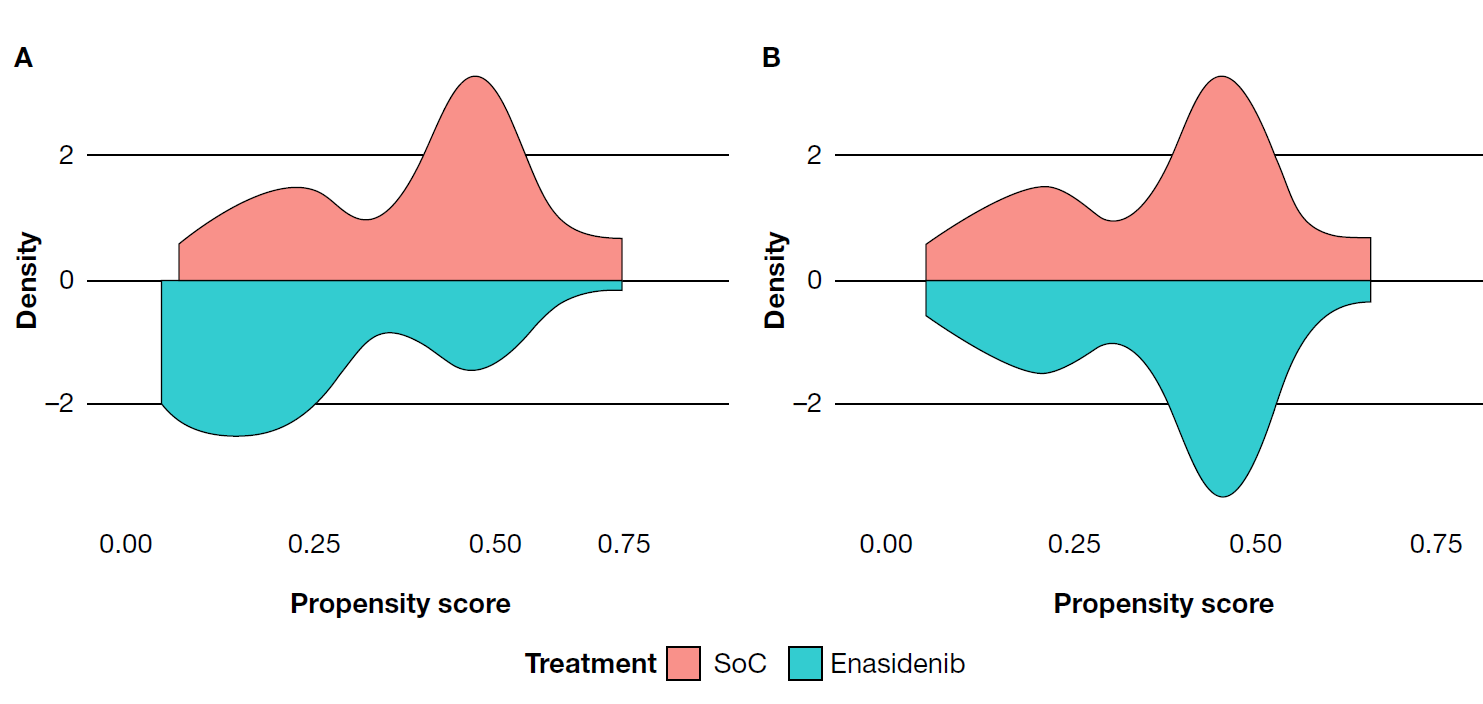


SoC, standard of care

The distributions in the sets of all patients (SoC: n = 80; enasidenib: n = 195) and matched patients (n = 78 patients in each group) are presented.
